# Vector-Borne and Zoonotic Diseases in Dogs and Cats

**DOI:** 10.3390/ani15111520

**Published:** 2025-05-23

**Authors:** Antonio Ortega-Pacheco, Matilde Jimenez-Coello

**Affiliations:** 1 Department of Animal Health and Preventive Medicine, Faculty of Veterinary Medicine, Autonomous University of Yucatan, Merida 97000, Yucatan, Mexico; 2 Regional Research Center “Dr. Hideyo Noguchi”, Biomedical Unit, Autonomous University of Yucatan, Merida 97000, Yucatan, Mexico; mjcoello@correo.uady.mx

Vector-borne and zoonotic diseases (VBZDs) remain some of the most dynamic and complex challenges in veterinary medicine, public health, and environmental sciences. Companion animals, particularly dogs and cats, have long been recognized as reservoirs of pathogens of zoonotic concern. Given the rapidly changing ecological, climatic, and socioeconomic conditions, understanding the shifting patterns of these diseases is imperative. This comprehensive volume brings together 11 peer-reviewed research articles originally published in a Special Issue of *Animals*, now consolidated into a thematic book offering a robust, multidisciplinary perspective on VBZDs in dogs and cats from a One Health perspective.

As we witness an unprecedented expansion in the distribution of vector species due to climate change, urbanization, deforestation, and globalization, the threat of emerging and re-emerging zoonoses grows. The One Health paradigm, which emphasizes the interconnectedness of human, animal, and environmental health, is the most suitable framework for addressing these complex dynamics [1,2]. Companion animals are increasingly central to these discussions, particularly dogs and cats, as their proximity to humans, vulnerability to vector-borne infections, and roles in transmission cycles render them both victims and facilitators of disease spread [3].

Leishmaniasis is a vector-borne parasitic disease of major concern in small animals and humans. Control of this infectious agent is crucial, and the search for candidate vaccines opens a wide area of research. This book opens with a study focusing on an innovative multi-epitope peptide vaccine candidate delivered via PLGA nanoparticles, which demonstrated promising immunogenic and protective effects in murine models. This research not only illustrates the advances in veterinary immunoprophylaxis but also offers hope for scalable, cost-effective preventive tools that could be adapted for field use. Complementing this, clinical evaluation of meglumine antimoniate in dogs with naturally acquired *Leishmania infantum* infection, despite potential adverse reactions, can achieve significant reductions in parasitic load and symptomatology, reinforcing its role in therapeutic protocols while underscoring the need for improved, less toxic alternatives. These studies are critical in regions where leishmaniasis remains endemic and where co-infection with other pathogens complicates clinical management.

Heartworm disease caused by *Dirofilaria immitis* is a parasitic cardiopulmonary infection with implications for both canine and feline health. This agent is widely distributed worldwide but is considered hyperendemic in some areas, such as the Canary Islands [4]. Using radiographic thoracic indices demonstrates that specific imaging markers can predict pulmonary hypertension, a serious sequela of advanced heartworm disease. Meanwhile, radiographic evaluations in cats highlight subtle *D. immitis*-induced vascular changes that precede overt clinical signs, revealing opportunities for earlier diagnosis and intervention. In hyperendemic regions, it is crucial to control *D. immitis* in dogs, so this volume presents an important review on the use of long-acting moxidectin, demonstrating its efficacy and benefits when preventative medicine is a problem for owners.

Chagas disease (American trypanosomiasis) is transmitted by vectors and caused by the protozoan *Trypanosoma cruzi*, although it is commonly associated with humans. Another study confirms that dogs are capable of developing significant cardiomyopathy, detectable through echocardiographic parameters [5]. These findings are particularly relevant in Latin America, where peridomestic transmission involving dogs is key in maintaining the life cycle of *T. cruzi* [6]. Another critical zoonotic bacterial infection examined in this volume is leptospirosis, whose global burden remains underestimated [7]. A study presented here underscores the value of region-specific antigens in serological assays, which significantly improve the sensitivity and specificity of ELISA tests. This is particularly relevant given that *Leptospira* spp. are highly diverse, and local strains often differ antigenically from reference laboratory strains.

Protozoan and nematode zoonoses have notably been reported. One article revisits the epidemiology of *Cryptosporidium* spp. and *Giardia duedenalis* in dogs and cats, where human assemblage strains demonstrate their potential cause of zoonotic transmission [8]. There is compelling molecular evidence that *G. intestinalis* assemblage E is present in symptomatic children in rural settings, implicating animal-to-human transmission and challenging established epidemiological assumptions [9]. This aligns with calls for a re-evaluation of host specificity and better integration of molecular diagnostics in surveillance systems. *Toxocara* spp. are highly relevant nematodes distributed worldwide. Their main hosts are dogs (*T. cani*) and cats (*T. cati*) [10], but they also have significant zoonotic potential with severe clinical consequences [11]. The systematic review and meta-analysis of *T. cati* presented in this book [12] describes its widespread prevalence and highlights the necessity for actions to mitigate the clinical manifestations in cats and the risk of zoonosis.

Trematode infections, particularly *Opisthorchis viverrini* and *Clonorchis sinensis*, have been reviewed in light of emerging evidence from Vietnam and Southeast Asia [13,14]. These parasites are known for their carcinogenic potential, particularly in relation to cholangiocarcinoma. However, conventional copro-parasitological diagnostics often fail to distinguish between these liver flukes and minute intestinal trematodes. The reviewed data stress the necessity for robust diagnostic alternatives such as genetic markers and the expansion of molecular tools in endemic settings where dogs and cats may be involved in the epidemiology of these parasites [15].

This book serves not only as a repository of current scientific evidence but also as a tool for veterinary practitioners, epidemiologists, policymakers, and students engaged in the control of zoonotic and vector-borne diseases. Its relevance spans endemic and non-endemic regions alike, given the increasingly transboundary nature of infectious disease emergence. We are grateful to the contributing authors for their high-quality work and to the editorial team of *Animals* for supporting the consolidation of this Special Issue into a dedicated book. We hope that this compilation will inspire further crossdisciplinary collaborations and inform more effective, evidence-based interventions in the field of zoonoses and vector-borne diseases in companion animals.
